# Ethyl acetate extract of germinated brown rice attenuates hydrogen peroxide-induced oxidative stress in human SH-SY5Y neuroblastoma cells: role of anti-apoptotic, pro-survival and antioxidant genes

**DOI:** 10.1186/1472-6882-13-177

**Published:** 2013-07-17

**Authors:** Nur Hanisah Azmi, Norsharina Ismail, Mustapha Umar Imam, Maznah Ismail

**Affiliations:** 1 Laboratory of Molecular Biomedicine, Institute of Bioscience, Universiti Putra Malaysia, 43400 UPM Serdang, Selangor, Malaysia; 2 Department of Nutrition and Dietetics, Universiti Putra Malaysia, 43400 UPM Serdang, Selangor, Malaysia

**Keywords:** Germinated brown rice, Antioxidant, Oxidative stress, Neuroprotective, SH-SY5Y

## Abstract

**Background:**

There are reports of improved metabolic outcomes due to consumption of germinated brown rice (GBR). Many of the functional effects of GBR can be linked to its high amounts of antioxidants. Interestingly, dietary components with high antioxidants have shown promise in the prevention of neurodegenerative diseases like Alzheimer’s disease (AD). This effect of dietary components is mostly based on their ability to prevent apoptosis, which is believed to link oxidative damage to pathological changes in AD. In view of the rich antioxidant content of GBR, we studied its potential to modulate processes leading up to AD.

**Methods:**

The total phenolic content and antioxidant capacity of the ethyl acetate extract of GBR were compared to that of brown rice (BR), and the cytotoxicity of both extracts were determined on human SH-SY5Y neuronal cells using 3-(4,5-Dimethylthiazol-2-yl)-2,5-diphenyltetrazolium bromide (MTT) Assay. Based on its higher antioxidant potentials, the effect of the GBR extract on morphological changes due to hydrogen peroxide (H_2_O_2_)-induced oxidative damage in human SH-SY5Y neuronal cells was examined using inverted light microscope and fluorescence microscope by means of acridine orange-propidium iodide (AO/PI) staining. Also, evaluation of the transcriptional regulation of antioxidant and apoptotic genes was carried out using Multiplex Gene Expression System.

**Results:**

The ethyl acetate extract of GBR had higher total phenolic content and antioxidant capacity compared to BR. The cytotoxicity results showed that GBR extract did not cause any damage to the human SH-SY5Y neuronal cells at concentrations of up to 20 ppm, and the morphological analyses showed that the GBR extract (up to 10 ppm) prevented H_2_O_2_-induced apoptotic changes in the cells. Furthermore, multiplex gene expression analyses showed that the protection of the cells by the GBR extract was linked to its ability to induce transcriptional changes in antioxidant (SOD 1, SOD 2 and catalase) and apoptotic (AKT, NF-Kβ, ERK1/2, JNK, p53 and p38 MAPK) genes that tended towards survival.

**Conclusions:**

Taken together, the results of our study showed that the ethyl acetate extract of GBR, with high antioxidant potentials, could prevent H_2_O_2_-induced oxidative damage in SH-SY5Y cells. The potential of GBR and its neuroprotective mechanism in ameliorating oxidative stress-related cytotoxicity is therefore worth exploring further.

## Background

Oxidative stress and apoptosis underlie the pathogenesis of several neurodegenerative diseases including Alzheimer’s disease (AD) [1,2]. Reactive oxygen species (ROS) produced in excess due to endogenous and/or exogenous stimuli would normally induce signal transduction that may result in mitochondrial dysfunction, cell death or cell survival [3]. The outcome of such insults on cells depends largely on the enormity of the insult and the ability of the cells to contain the insults or remove accumulating ROS. The brain cells are normally sensitive to the effects of ROS because they are a nidus for peroxidisable molecules, and because of their peculiar energetic demands [4]. In AD, ROS starts to accumulate in neurons before clinically evident signs and symptoms of the disease can be detected [1,4]. Studying mechanisms by which neurons cope with oxidative insults, therefore, serve as important avenues to evaluate potential preventive or therapeutic agents for AD development. When ROS accumulate, oxidative damage is normally prevented by induction of protective factors like antioxidants, which may not be effective if the insult is too overwhelming. In such cases, apoptotic mechanisms set in to remove neurons deemed unsalvageable [1,5]. Loss of neurons through these apoptotic deaths results in severe morphological and functional deficits, which manifest with progressive memory and cognitive decline.

Interestingly, the signal transduction mechanisms involved in neuronal survival or death are closely related. In fact, some shared mechanisms between the two extremes have been reported [3]. Specifically, the tumor suppressor gene p53 is reportedly activated by several oxidative insults including hydrogen peroxide (H_2_O_2_). In response to its activation, other pathways are induced, mostly involving mitogen-activated protein kinases (MAPKs) like JNK and p38 [1]. The overall response of neurons to stimuli, however, depends on which pathways are predominantly regulated. For example, induction of the MAPKs (JNK and p38) is reportedly able to activate heme oxygenase-1 (HO-1), which counteracts the effects of ROS [6]. Also, induction of cyclo-oxygenase-II (COX-II) has been shown to ameliorate the harmful effects of ROS through its negative effects on p53 [7-9]. These complex interactions between pro-apoptotic and pro-survival pathways suggest that the neurons constantly work to prevent cell death and eventually morphological and functional defects on the brain except in extreme conditions, when they resort to apoptosis. The result of this damage is the development of senile plaques composed of primarily aggregated amyloid fibrils, and other pathological changes of AD, which also propagate the disease [5]. However, endogenous production of antioxidant defenses like superoxide dismutases (SODs), activated by the presence of ROS, could initially boost the cells’ ability to reduce ROS and remove the underlying stimuli [3]. This line of defense (antioxidants) can be potentiated through exogenous supplementation. Dietary antioxidants have been shown to block the pathways involved in apoptosis and eventual neuronal cell death, and hence their potential role in preventing or at least delaying the development of AD [10]. Curcumin, vitamins A, C and E, and flavonoids are notable examples of such antioxidants [10-12].

The promising role of antioxidants in preventing and/or delaying the development and progression of AD has generated interest in the search for other potent antioxidants with similar potential effects. Germinated brown rice (GBR) has been reported to have high antioxidant capacity [13]. It was also reported to improve antioxidant status in type 2 diabetes [14] and protect neuronal cells from oxidative stress-induced cytotoxicity [15,16]. This property of GBR is thought to be contributed by the potentiation of its bioactive compounds during the process of germination of brown rice (BR) [17]. Thus we studied the effects of a GBR extract on H_2_O_2_-induced oxidative stress in human SH-SY5Y neuronal cells and the transcriptional regulation of antioxidant and apoptotic genes. Human SH-SY5Y are derived from its parent SK-N-SH neuroblastoma cells. Terminally differentiated human SH-SY5Y neuroblastoma cells are often used as an in vitro model to study neurodegenerative diseases because of their ability to respond in a similar manner to mature human neurons, when exposed to chemical agents in vitro [18,19]. We hypothesized that in view of GBR’s higher content of antioxidant bioactives and better effects on oxidative stress than BR, its ethyl acetate extract could protect against H_2_O_2_-induced oxidative stress. Also, because apoptosis is important in oxidative stress-induced neurotoxicity, an understanding of its transcriptional regulatory mechanisms would provide a better insight into how GBR and its extracts exert their neuroprotective effects. This may broaden the understanding of the potential role of GBR in regulation of processes involved in neurodegenerative diseases and other closely related conditions.

## Methods

### Materials

All solvents were of analytical grade and were purchased from Merck (Darmstadt, Germany). Potassium persulphate (K_2_S_2_O_8_), sodium carbonate (Na_2_CO_3_), 2,2’-azino-bis[3-ethylbenzothiazoline-6-sulphonic acid] (ABTS) reagent, di[phenyl]-[2,4,6-trinitrophenyl] iminoazanium (DPPH) reagent, Folin–Ciocalteu reagent, Trolox standard, gallic acid standard, Ham’s nutrient mixture F-12, Minimum Essential Eagle’s medium, fetal bovine serum, antibiotics and 3-(4,5-Dimethylthiazol-2-yl)-2,5-diphenyltetrazolium bromide (MTT) powder were purchased from Sigma-Aldrich (St. Louis, MO, USA). Other cell culture materials were purchased from BD Biosciences (NJ, USA). H_2_O_2_ and sodium hypochlorite (NaOCl) were from Bendosen Laboratory Chemicals (Selangor, Malaysia) and from Dexchem Industries Sdn. Bhd. (Penang, Malaysia), respectively. The GenomeLab™ GeXP Start Kit was purchased from Beckman Coulter Inc. (Miami, FL, USA), and the Total RNA Isolation kit was from RBC Bioscience Corp. (Taipei, Taiwan). Magnesium chloride (MgCl_2_) and DNA Taq polymerase were purchased from Thermo Fisher Scientific (Pittsburgh, PA, USA).

### Germination of brown rice and preparation of extract

PadiBerasNasional (BERNAS) factory, Sri Tiram Jaya, Selangor provided the BR of Malaysian variety (MR219 and MR220) used in this study. BR was germinated as reported in an earlier publication [14]. Briefly, BR was soaked in 0.1% NaOCl(1:5, w/v) and 0.5% H_2_O_2_ (1:5, w/v) for 30 min and 6 h respectively, and incubated at 37°C for 18 h. Germination was shown by sprouting, and the final moisture content was 8-11% after drying at 50°C.

GBR and BR were dried and ground using a grinder. Extraction of GBR and BR were respectively carried out using ethyl acetate. Briefly, 5 g of GBR and BR powders were dissolved in 20 mL of ethyl acetate. The mixtures were sonicated for 1 h. The extracts were then filtered through Whatman filter paper No. 1 and the entire extraction process was repeated twice on the residue obtained from the filtration process. The filtrates were pooled and solvent was removed from the filtrates under reduced pressure (Rotavapor R210, Buchi, Postfach, Flawil, Switzerland). Finally, the extracts were cooled in a desiccator and kept at −80°C until further analyses.

### DPPH free radical and ABTS radical cation scavenging assays

The abilities of GBR and BR extracts to scavenge DPPH free radical were determined according to the method described by Chan et al., 2012 [20]. Briefly, 4.2 mg of DPPH powder was dissolved in 50 mL of 100% methanol (0.2 mM). A Trolox stock solution was prepared as a standard. GBR and BR extracts were reacted with 195 μL of DPPH methanolic solution in 96-well microtitre plates. After 60 minutes of incubation, the absorbance values of the samples were read at 540 nm using a microplate reader (Opsys MR™ 96-well microplate reader, Dynex Technologies, VA, USA). DPPH scavenging activities of the extracts/Trolox standard were calculated using the following equation:-

$$\mathrm{DPPH}\;\mathrm{Scavenging}\;\mathrm{Activity}=\frac{\mathrm{Ab}s_{\mathrm{negative}\;\mathrm{control}}-\mathrm{Ab}s_{\mathrm{extract}/s\tan\mathrm{dard}}}{\mathrm{Ab}s_{\mathrm{negative}\;\mathrm{control}}}\times 100\%$$

ABTS radical scavenging activities of the extracts were determined according to the method reported by Kim et al., 2010 [21]. Briefly, 6.62 mg of K_2_S_2_O_8_ was dissolved in 10 mL of distilled water to prepare a solution of 2.45 mM.Then, 7 mM ABTS was prepared by dissolving 38.4 mg in 10 mL distilled water. The two solutions were mixed and incubated in the dark for 16 h prior to use. The mixture was diluted with distilled water until a spectrophotometric absorbance of 0.700 ± 0.005 at 735 nm was obtained. Next, 100 μL of samples/Trolox standard were reacted with 900 μL of the diluted ABTS solution and vortexed. The absorbance was read at 734 nm. ABTS radical cation scavenging activity was calculated as the percentage reduction in absorbance.

### Total Phenolic content analysis

Total phenolic contents (TPCs) of GBR and BR extracts were determined using Folin-Ciocalteu method. Briefly, 10 mg of the extracts were dissolved in 1 mL of methanol. The solutions (0.1 mL) were individually mixed with 2.5 mL of 10-fold diluted Folin-Ciocalteau reagent, and 2.0 mL of 7.5% Na_2_CO_3_. After incubation at 40°C for 60 min, the absorbance of the reaction mixture was measured at 760 nm using a spectrophotometer (Pharmaspec UV-1700, Shimadzu, Kyoto, Japan). Gallic acid was used as a standard and TPC of the extract was expressed in mg gallic acid equivalents (mg GAE/g extract).

### Cell culture

The human neuroblastoma SH-SY5Y cells were grown in complete culture medium containing mixture (1:1) of Minimum essential Eagle’s medium and Ham’s nutrient mixture F-12, which was supplemented with 10% fetal bovine serum, 1% MEM non-essential amino acids and 50 μg/mL gentamicin. Cells were maintained at 37°C under 5% CO_2_ and 95% air. Dimethyl sulfoxide (DMSO) concentration was maintained at 0.1% for all cell culture assays.

### MTT assay

The abilities of GBR and BR to protect SH-SY5Y cells from H_2_O_2_ were determined by MTT assay, which is a potential indicator of cell viability. SH-SY5Y cells were seeded into 96-well culture plates at a density of 2 × 10^5^ cells/mL and were allowed to attach. After 2 days, cells were differentiated with retinoic acid (10 μM) for 6 days prior to treatment. To examine the possible toxic effects, the cells were treated with GBR and BR over a concentration range of 1–30 ppm for 24 h. For the determination of neuroprotective effects, cells were pretreated with the respective extracts diluted in serum-free medium for 24 h and then challenged with 250 μM H_2_O_2_, which is the IC_50_ of H_2_O_2_[15]_,_ for another 1 h. MTT was added to all the wells and allowed to incubate in the dark at 37°C for 4 h. The amount of MTT formazan product was determined by measuring absorbance at 540 nm using a Microplate reader (Opsys MR, Thermo Labsystems, Franklin, MA, USA). All the MTT assays were performed in triplicate.

### Morphological analysis using inverted light microscope

SH-SY5Y cells were seeded into 6-well culture plates at a density of 2 × 10^5^ cells/mL and were allowed to attach. After 2 days, cells were differentiated with retinoic acid (10 μM) for 6 days prior to treatment. Cells were treated with GBR at concentrations of 1 and 10 ppm for 24 h before exposing them to 250 μM H_2_O_2_ for another 1 h. After the incubation period, cultures were observed under phase contrast, using a 40× objective in an inverted microscope (Nikon ECLIPSE TS100, Nikon Corporation, Tokyo, Japan) and then photographed using a digital camera (Nikon DS-Fi1, Nikon Corporation, Tokyo, Japan) and the image acquisition software NIS Elements D 3.0 version. Multiple independent images were taken.

### Acridine orange and propidium iodide (AO/PI) staining using fluorescence microscope

SH-SY5Y cells were seeded, treated with GBR and 250 μM H_2_O_2_ as previously described. After the incubation period, the growth medium was discarded. Cells were then stained with the dye mixture (10 μL of 1 mg/mL AO and 10 μL of 1 mg/mL PI). Stained cells were examined using an inverted fluorescence microscope (Olympus, Tokyo, Japan). Multiple independent images were taken.

### Multiplex gene expression analysis

#### RNA extraction

At the end of the experiment, SH-SY5Y cells treated with GBR extract were washed with phosphate-buffered saline (PBS), and RNA isolated using Total RNA Isolation kit (RBC Bioscience Corp., Taiwan) according to the manufacturer’s protocol. RNA concentration was determined using NanoDrop spectrophotometer (Thermo Scientific Nanodrop, NanoDrop Technologies, Wilmington, DE, USA). The ratios of A260/230 and A260/280 were used to indicate the purity of extracted total RNA.

#### Primer design

Primers were designed on the GenomeLabeXpress Profiler software using *Homo sapien* sequence adopted from the National Center for Biotechnology Information GenBank Database [22]. The genes of interest, housekeeping genes and internal control are shown on Table 1. The forward and reverse primers had universal tag sequences in addition to nucleotides that were complementary to the target genes. Primers were supplied by First Base Ltd. (Selangor, Malaysia), and diluted in 1 X TE Buffer to a concentration of 500 nM for reverse primers and 200 nM for forward primers.

**Table 1:** Gene name, accession number, reverse and forward primer sequences used in GeXP multiplex gene expression analysis

**Gene name**	**Accession number**	Primer sequences* with universal tags (underlined)	
		**Forward**	**Reverse**
GAPDH^a^	NM_002046	AGGTGACACTATAGAATAAAGGTGAAGGTCGGAGTCAA	GTACGACTCACTATAGGGAGATCTCGCTCCTGGAAGATG
KanR^b^	-	AGGTGACACTATAGAATAATCATCAGCATTGCATTCGATTCCTGTTTG	GTACGACTCACTATAGGGAATTCCGACTCGTCCAACATC
Hyaluronidase^a^	AJ000099	AGGTGACACTATAGAATACAGCAGTTCATGCTGGAGAC	GTACGACTCACTATAGGGACCAGGTAGACAGACGGGAAG
18 sRNA^a,#^	M10098	AGGTGACACTATAGAATAGGAGTGGAGCCTGCGGCTTAA	GTACGACTCACTATAGGGATAGCATGCCAGAGTCTCGTT
ACTB^a^	NM_001101	AGGTGACACTATAGAATAGATCATTGCTCCTCCTGAGC	GTACGACTCACTATAGGGAAAAGCCATGCCAATCTCATC
AKT	NM_001014431	AGGTGACACTATAGAATAGAGGAGATGGACTTCCGGTC	GTACGACTCACTATAGGGAAGGATCTTCATGGCGTAGTAGC
p53	NM_001126117	AGGTGACACTATAGAATAGGGGAGCAGGGCTCA	GTACGACTCACTATAGGGAAAAATGGCAGGGGAGGG
JNK	NM_139046	AGGTGACACTATAGAATACAGAAGCTCCACCACCAAAGAT	GTACGACTCACTATAGGGAGCCATTGATCACTGCTGCAC
ERK1/2	NM_002745	AGGTGACACTATAGAATAGGAGCAGTATTACGACCCGA	GTACGACTCACTATAGGGAGATGTCTGAGCACGTCCAGT
p38 MAPK	NM_001315	AGGTGACACTATAGAATATTCAGTCTTTGACTCAGATGCC	GTACGACTCACTATAGGGAGTCAGGCTTTTCCACTCATCT
SOD 1	NM_000454	AGGTGACACTATAGAATATCATCAATTTCGAGCAGAAGG	GTACGACTCACTATAGGGA TGCTTTTTCATGGACCACC
SOD 2	NM_000636	AGGTGACACTATAGAATACATCAAACGTGACTTTGGTTC	GTACGACTCACTATAGGGACTCAGCATAACGATCGTGGTT
Catalase	NM_001752	AGGTGACACTATAGAATAGAAGTGCGGAGATTCAACACT	GTACGACTCACTATAGGGA ACACGGATGAACGCTAAGCT

*Based on the *Homo sapien* gene sequences adopted from the National Center for Biotechnology Information GenBank Database [22]. ^a^ Housekeeping genes; ^b^ Internal control; ^#^ Normalization gene.

#### Reverse transcription and polymerase chain reaction (PCR)

Reverse transcription (RT) and multiplex PCR of RNA samples (50 ng/μL) were carried out in XP Thermal Cycler (BIOER Technology, Hangzhou, China) according to GenomeLab™ GeXP Start Kit (Beckman Coulter, Inc, Miami, FL, USA). Briefly, RT reaction mixture was prepared using RNA sample (1 μL each), 4 μL of 5X RT Buffer, 2 μL of RT Reverse Primers, 1 μL of KanR, 1 μL of Reverse Transcriptase and 11 μL of DNAse/RNase free water. cDNA was synthesized according to the reaction protocol: 48°C for 1 min, 42°C for 60 min, 95°C for 5 min and 4°C hold. Also, 9.3 μL of each cDNA was mixed with 10.7 μL of PCR reaction mixture consisting of 5X PCR Buffer, 25 mM MgCl_2_, PCR Forward Primer Plex, and Thermo-Start DNA polymerase. Amplification conditions were 95°C for 10 min, followed by 34 cycles of 94°C for 30 sec, 55°C for 30 sec, 70°C for 1 min and 4°C hold.

#### GEXP data analysis

PCR products (1 μL each) from the above reactions were mixed with 38.5 μL of sample loading solution and 0.5 μL of DNA size standard 400 (Beckman Coulter, Inc, Miami, FL, USA) in a 96-well sample loading plate and analyzed on the GeXP machine (Beckman Coulter, Inc, Miami, FL, USA). The results from the machine were analyzed using the Fragment Analysis module of the GeXP system software and then imported onto the analysis module of eXpress Profiler software. Normalization was performed with 18 sRNA according to manufacturer’s instructions.

### Statistical analysis

All data are presented as mean ± SD. The data was evaluated by one-way ANOVA using Statistical Package for Social Sciences software, version 20 (SPSS Inc., Chicago, IL). Differences between the means were assessed using Tukey’s multiple comparisons and Student’s *t*-test. Statistical significance was considered at *p* < 0.05.

## Results and discussion

### DPPH free radical and ABTS radical cation scavenging activities and TPCs of GBR and BR

As can be recalled, GBR has higher antioxidant capacity than BR, due to its higher bioactive compounds including antioxidants [17]. As shown on Table 2, the antioxidant capacities of the ethyl acetate extracts used in this study suggest that GBR may have superior functional effects than BR. In addition, the TPCs of GBR and BR extracts were calculated using the linear standard curve, y = 0.0144x + 0.0267 (R^2^ = 0.9991), and were found to be 17.01 ± 1.27 and 10.81 + 0.24 mg GAE/g extract, respectively. These findings corroborate earlier reports on antioxidant capacities of GBR [23]. The role of antioxidants in prevention of apoptosis and other mechanisms that promote AD development is well established [10-12]. Through direct scavenging and/or activation of pro-survival mechanisms, antioxidants could prevent or at least delay the development of AD. The bioactive compounds responsible for the functional effects of GBR are still the subject of debate. However, multiple bioactive compounds may be responsible for any given effects due to GBR. We have reported a higher antioxidant capacity for methanolic extract of GBR compared to BR [13], while in two other studies we have demonstrated that the ethanolic extracts of GBR possessed higher antioxidant capacities [14,15]. These data indicate the abundance of antioxidants in GBR due to potentiation of bioactive compounds during germination. In view of the reports on anti-apoptotic effects of dietary components with high antioxidant potentials in AD, we hypothesized that our GBR type could regulate apoptosis and potentially prevent neuronal death.

**Table 2:** DPPH and ABTS antioxidant assays and total phenolic content for germinated brown rice (GBR) in comparison to brown rice (BR)

**Extract**	DPPH free radical scavenging assay^*,#^	**ABTS scavenging activity (mg TEAC/g extract)**^**#**^	**Total phenolic content (mg GAE/g extract)**^**#**^
GBR	2.32 + 0.04 ^**^	23.89 + 0.24 ^**^	17.01 + 1.27 ^**^
BR	10.37 + 0.47	3.59 + 0.09	10.81 + 0.24

^*^Expressed as IC_50_ (mg/ml), calculated from Trolox standard curve (y = 0.0087x + 4.587, r^2^ = 0.9957), with smaller values signifying higher antioxidant capacity. ^#^Values expressed as mean ± standard deviation. ^**^ Indicates statistical significance at p < 0.05 compared to BR for each assay represented in a column. ABTS, 2,2′-azino-bis[3-ethylbenzothiazoline-6-sulphonic acid]; DPPH, di[phenyl]-[2,4,6-trinitrophenyl]iminoazanium.

### MTT assay

**Figure 1:**
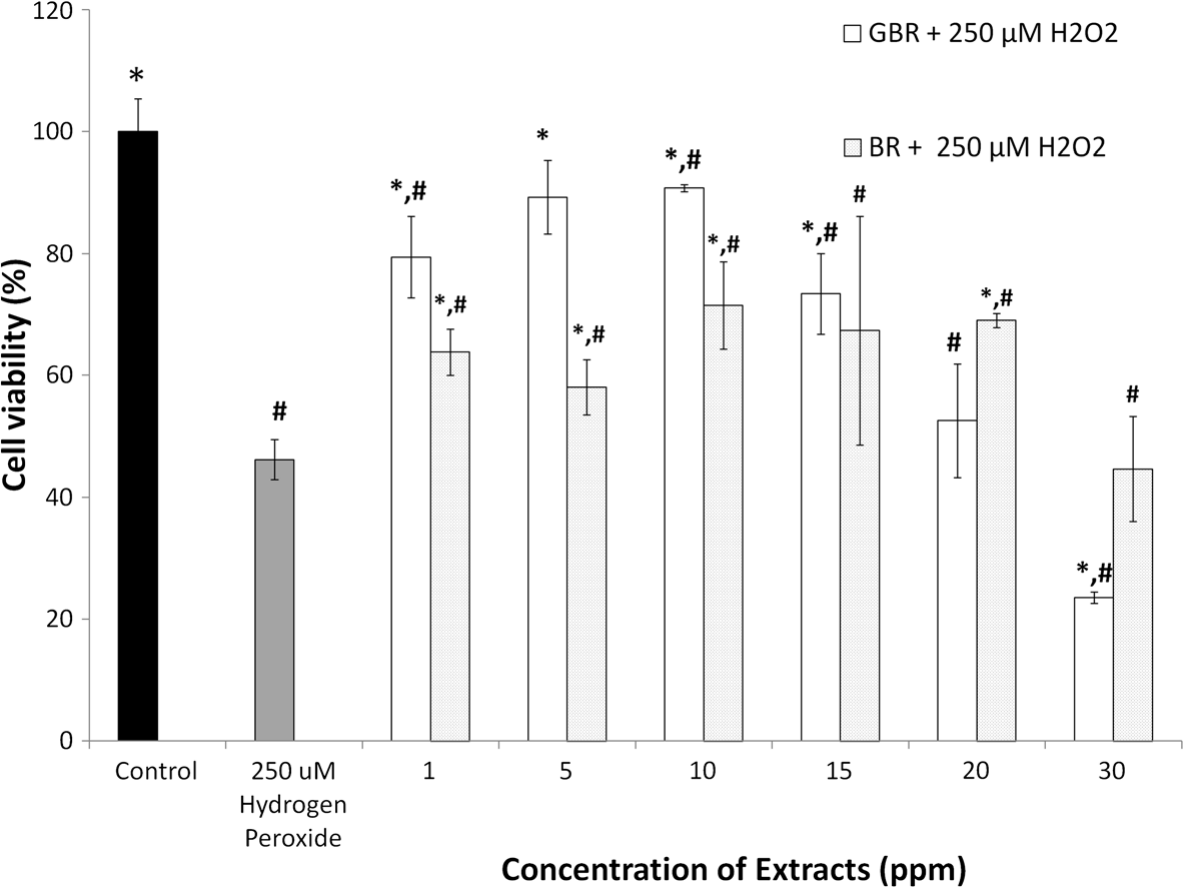
**Cell viabilities of SH-SY5Y cells after pretreatment with ethyl acetate extracts of germinated brown rice (GBR) and brown rice (BR), followed by hydrogen peroxide (H**_**2**_**O**_**2**_**); Cells were pretreated with GBR and BR extracts individually over concentration of 1–30 ppm for 24 h and subsequently incubated with or without 250 μM H**_**2**_**O**_**2**_**for 1 h.** Results are expressed as mean ± SD, *p < 0.05 *versus* H_2_O_2_. # p< 0.05 *versus* control.

To determine the effect of our antioxidant-rich extract on the viability of SH-SY5Y cells, we treated the cells with concentrations between 1–30 ppm. As reported in our earlier publication [15], we determined the IC_50_ of H_2_O_2_ to be 250 μM, hence it was used to challenge the cells after treatment with the ethyl acetate extracts. H_2_O_2_ is believed to induce intracellular defense mechanisms that tend towards production of more endogenous antioxidant, but when overwhelming, it leads to apoptosis and cell death [1]. In the presence of an exogenous antioxidant, the endogenous antioxidants get support in ameliorating the H_2_O_2_-induced oxidative damage [3,4]. As shown on Figure 1, concentrations of between 1 and 15 ppm of the ethyl acetate extract of GBR improved cell viability better than those of BR, likely due to its higher amounts of antioxidants. This is supported by earlier findings that dietary components with higher antioxidant capacities will promote neuronal cell survival [12]. However, the figure shows that at concentrations between 15 and 30 ppm of the GBR extract, cell viability reduced drastically compared to BR. This may have been due to suppression of endogenous antioxidant and other defenses that leave neurones vulnerable to more injury leading to a hormetic response. Under normal conditions, oxidative insults induce stress proteins and endogenous antioxidants that try to neutralize the stimuli. The presence of exogenous antioxidants potentiates these responses but when in excess, they could have a negative effect on endogenous cellular stress response, thereby increasing the sensitivity of the cells to stimuli and easily leading to cell death [24]. For our subsequent experiments, therefore, 1 and 10 ppm were used.

### Morphological analysis using inverted light microscope

**Figure 2:**
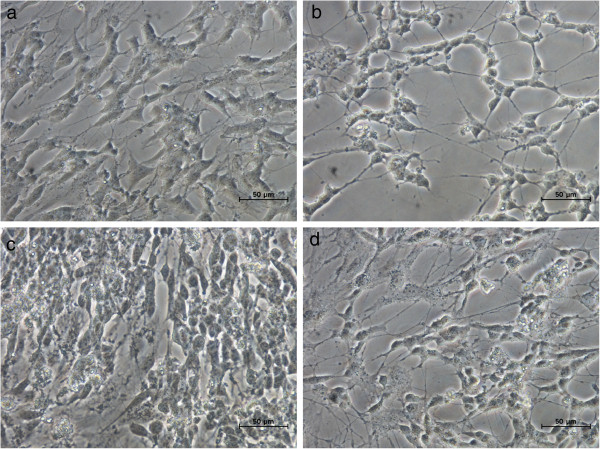
**Light micrographs of human SH-SY5Y cells.** Bar = 50 μM. SH-SY5Y cells viewed under light microscope, 40 X magnification; **(a)** Untreated control cells. **(b)** Treatment with250 μM H_2_O_2_ for 1 h. **(c)** Cells pretreated with 1 ppm of GBR extract and subsequent treatment with 250 μM H_2_O_2_ for 1 h. **(d)** Cells pretreated with 10 ppm of GBR extract and subsequent treatment with 250 μM H_2_O_2_ for 1 h.

Morphology of untreated and treated SH-SY5Y cells was observed under light microscope, to evaluate apoptotic features of the cells. From Figure 2, it can be seen that untreated control cells showed normal appearance of SH-SY5Y, which were elongated and flattened with axon-like outgrowths, while the H_2_O_2_-treated cells were shrunken, rounded, shiny and condensed, and showed surface blebs and disruption of the neurites [25]. The morphological analysis of the cells treated with the GBR extract showed that the H_2_O_2_-induced apoptotic changes were not present. This shows that the GBR extract effectively protected the cells from the oxidative damage caused by H_2_O_2_.

### Acridine orange (AO)/ propidium iodide (PI) staining

**Figure 3:**
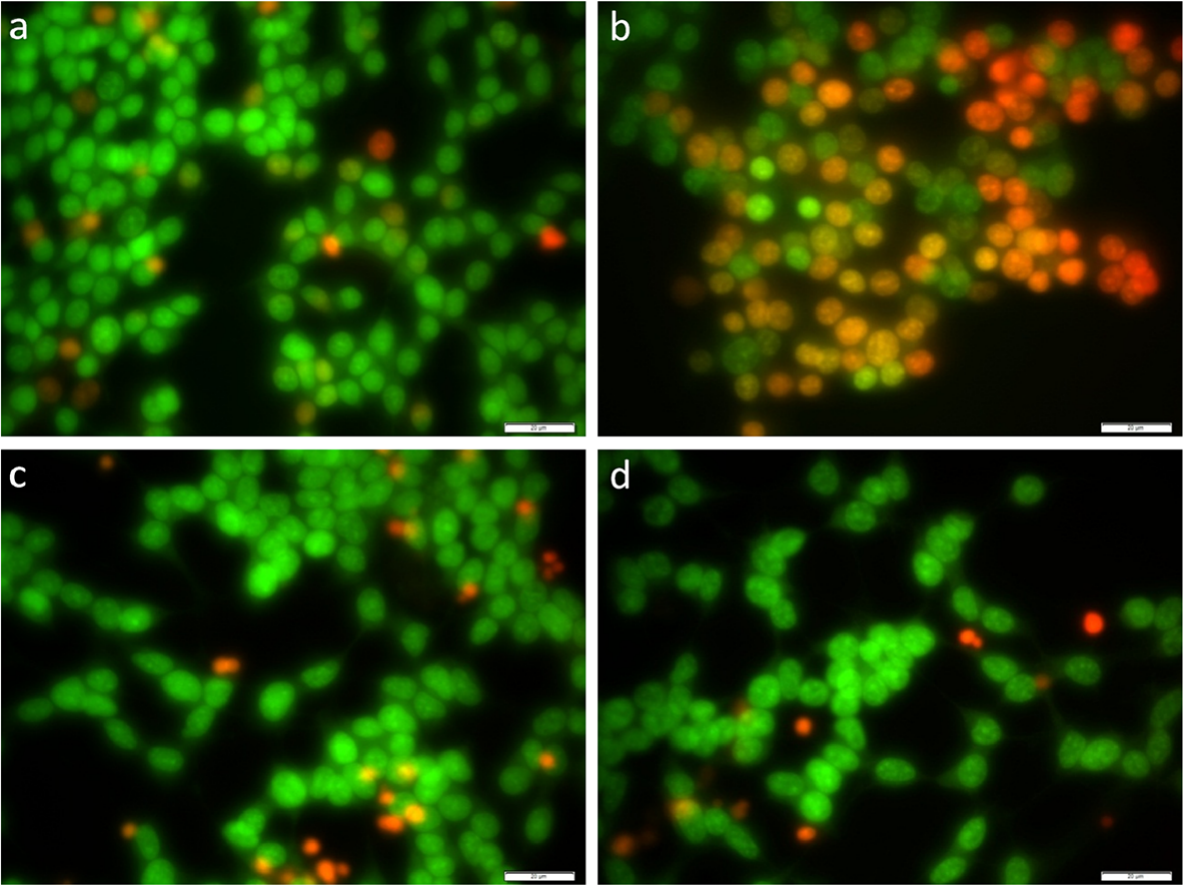
**Fluorescent micrographs of acridine orange (AO, green) and propidium iodide (PI, red) double-stained human SH-SY5Y cells viewed under fluorescence microscope.** Bar = 20 μM; **(a)** Untreated control cells. **(b)** Treatment with 250 μM H_2_O_2_ for 1 h. **(c)** Cells pretreated with 1 ppm of GBR extract and subsequent treatment with 250 μM H_2_O_2_ for 1 h. **(d)** Cells pretreated with 10 ppm of GBR extract and subsequent treatment with 250 μM H_2_O_2_ for 1 h.

Fluorescent microscopy was carried out to observe any morphological changes and apoptotic features of normal untreated and treated SH-SY5Y cells by means of AO and PI staining. AO is membrane-permeable and stains the cell nuclei green, indicating the cells are viable. On the other hand, the intercalating dye PI is membrane- impermeable, thus only taken up by non-intact cells and stains the nuclei red. As shown in Figure 3, the nuclei of untreated normal cells are stained green and showed normal structure, while H_2_O_2_-treated cells are stained red and orange, representing the hallmark of apoptosis. As for the GBR-treated cells, majority of the cells were stained green, showing normal appearance of intact cells.

### Effects of ethyl acetate extract of GBR on mRNA levels of antioxidant genes

**Figure 4:**
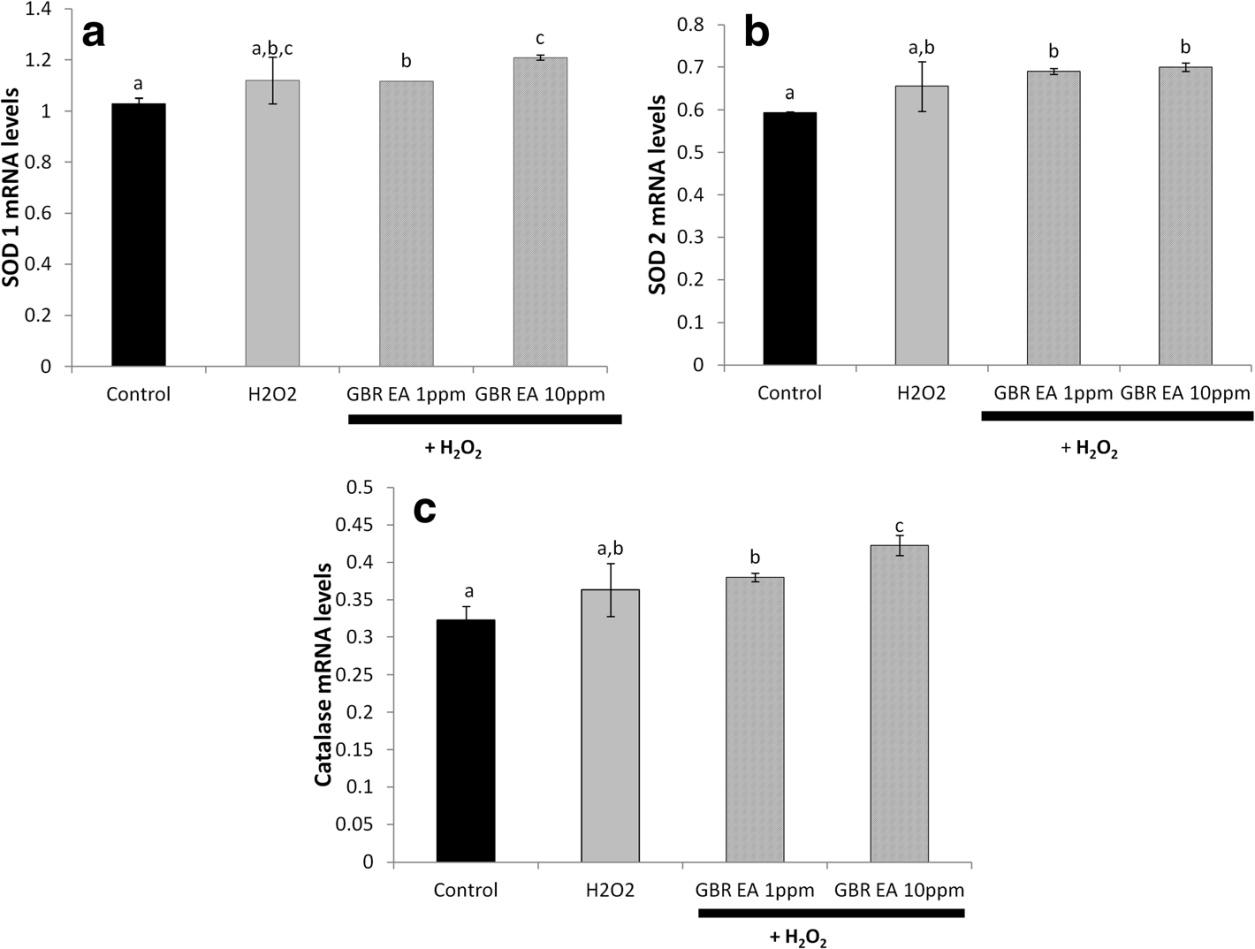
**Expression levels of the superoxide dismutase (SOD) 1, SOD 2 and catalase genes in SH-SY5Y cells.** mRNA levels of **(a)** the SOD 1, **(b)** the SOD 2, and **(c)** the catalase genes following treatment with 1 and 10 ppm of GBR extract and subsequent treatment with 250 μM H_2_O_2_, in comparison to untreated and 250 μM H_2_O_2_-treated controls. Bars represent means of groups (n=3) and error bars represent SDs. Different letters on any 2 bars representing each gene indicate statistically significant difference (p < 0.05) between the groups.

The mRNA levels of three antioxidant genes (catalase, SOD 1 and SOD 2) and six apoptotic/oxidative stress-related genes were studied using Multiplex GeXP genetic analysis system, with KanR as the internal control. The complete list of target and housekeeping genes is shown on Table 1. As shown in Figure 4, the treatment with 250 μM H_2_O_2_upregulated the mRNA levels of all antioxidants, although only SOD 2 was significantly higher than that of untreated cells. The treatments with GBR extract at concentrations of 1 and 10 ppm, followed by subsequent treatment with 250 μM H_2_O_2_upregulated the expression of the three antioxidant genes in comparison to H_2_O_2_-treated controls and normal untreated cells. In the case of SOD 1, the GBR extract upregulated the gene significantly higher than untreated controls in a dose-dependent manner, but did not show significantly different expression levels in comparison to H_2_O_2_- treated controls. The mRNA levels of SOD 2 were also higher in the GBR-treated cells compared to normal untreated cells and H_2_O_2_-treated controls, although there was no significant difference between H_2_O_2_-treated and GBR-treated cells. The mRNA levels of catalase were similar to SOD 1, in which the expression due to GBR treatment was found to be higher than in normal untreated cells but not significantly higher than in H_2_O_2_-treated cells.

The expression patterns of the antioxidant genes due to H_2_O_2_treatment is in agreement with findings that H_2_O_2_ normally induces antioxidant defenses, especially SODs, in cells [1]. The upregulation of antioxidant defenses is an attempt to boost endogenous antioxidants to prevent oxidative damage on cells [3,4]. In addition, exogenous antioxidants may potentiate the expression of the antioxidant genes as seen in Figure 4. The implication of the GBR treatments is that the potentiation of the antioxidant gene expression by the GBR extracts would confer more protection to the cells. This upregulation of antioxidant genes is similar to what we have reported previously in diabetic rats due to consumption of GBR [14], and in Hep-G2 cells due to different extracts of GBR [26].

### Effects of ethyl acetate extract of GBR on mRNA levels of p53 and MAP kinases (JNK, ERK1/2 and p38), AKT and nuclear factor kappa β (NF-Kβ)

**Figure 5:**
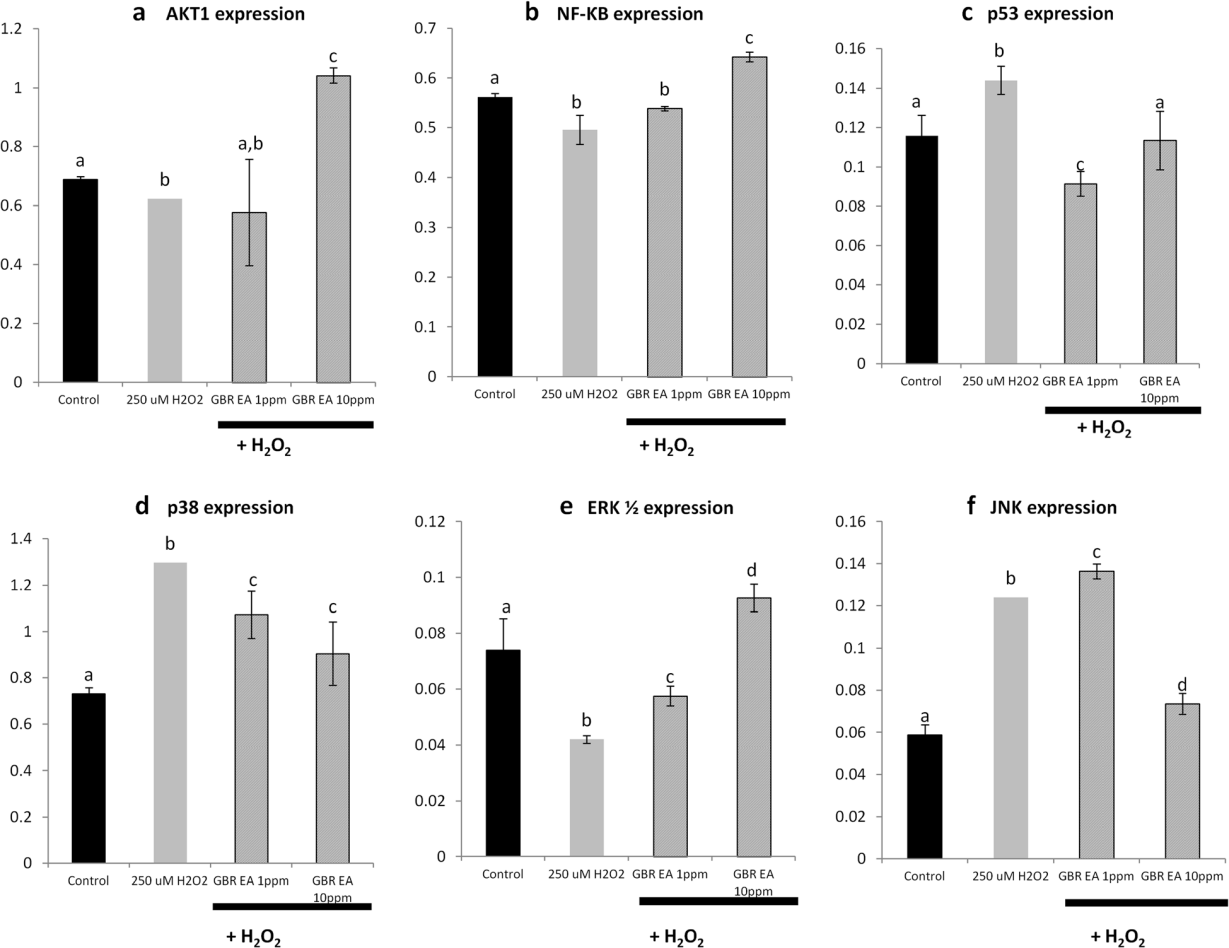
**Expression of AKT, NF-Kβ, p53, p38 MAPK, ERK1/2 and JNK genes in SH-SY5Y cells.** mRNA levels of **(a)** the AKT, **(b)** the NF-Kβ, **(c)** the p53, **(d)** the p38 MAPK, **(e)** the ERK1/2, and **(f)** the JNK genes following treatment with 1 and 10 ppm of GBR extract and subsequent treatment with 250 μM H_2_O_2_, in comparison to untreated and 250 μM H_2_O_2_-treated controls. Bars represent means of groups (n=3) and error bars represent SDs. Different letters on any 2 bars representing each gene indicate statistically significant difference (p < 0.05) between the groups.

H_2_O_2_ is known to induce oxidative damage to neuronal cells through induction of apoptotic mechanisms. These involve activation of transcriptional factors and signal transducers like p53 and MAPKs [1]. In the current study, we evaluated the possible apoptotic mechanisms modulated by the ethyl acetate extract of GBR in improving neuronal cell survival. Figure 5 shows the effects of the GBR extract on p53, MAPKs, AKT and NF-Kβ. H_2_O_2_ significantly upregulated p53, p38 and JNK, while it downregulated ERK1/2, AKT and NF-Kβ, compared to untreated controls. These factors may all mediate cell survival or death [3], and it appears that their patterns of expression tended towards activation of apoptotic mechanisms [1,4]. Upregulation of p53, p38 and JNK are normal responses to ROS, although they could also mediate protective mechanisms. However, it is the downregulation of ERK1/2, AKT and NF-Kβ that suggest likely activation of a death signal, because their upregulation have all been linked to cell survival through activation of COX-II [1,27]. Their downregulation by H_2_O_2_ in the current study suggests that neuronal cells treated with H_2_O_2_ alone were inclined towards apoptosis rather than cell survival.

Treatment with GBR extract (1 and 10 ppm) prior to the H_2_O_2_ insult showed signs of anti-apoptosis towards the SH-SY5Y cells by upregulating the expression of AKT, NF-Kβ as well as ERK1/2. Additionally, the GBR treatment resulted in suppression of the p53, p38 and JNK compared to the H_2_O_2_-treated control. The effects of different doses of GBR extract (1 and 10 ppm) on p38 MAPK and JNK gene expressions were found to be in a dose-dependent manner as well, in which higher concentration showed higher suppression of the genes. Likewise, the treatment of GBR extract at 1 ppm also significantly lowered the expression of p53 gene. However, the expression of the gene following treatment with 10 ppm of the extract appeared to have slightly increased, but not significantly different from untreated control. Increase in mRNA levels of AKT, NF-Kβ and ERK1/2, and decrease in those of JNK, p38 and p53 suggest that the GBR extract may have anti-apoptotic and pro-survival effects on neuronal cells. Activation of COX-II by AKT and NF-Kβ is known to have a negative effect on the transcriptional level and activity of p53, which could eventually lead to lower transcription of JNK and p38, since p53 modulates the activities of the latter two [1,27]. In fact, upregulation of the AKT gene has been shown to be a very important mechanism in regulating p53-induced apoptosis [28], signifying its importance in the prevention of apoptosis in the current study.

Transcriptional regulation of genes that regulate apoptosis like JNK and NF-Kβ are mechanisms by which dietary components have been shown to modulate the apoptosis and survival of neuronal cells and eventually the risk of AD [11]. Neuronal death due to oxidative stress mediated by apoptosis largely contributes to the pathological changes and eventual symptomatology of AD and other neurodegenerative diseases. The abilities of dietary components to potentially prevent or at least retard the progression of these diseases have been linked to their abilities to prevent neuronal death and enhance cell survival through prevention of apoptosis. In this regard, dietary components, like flavonoids and curcumin, with high antioxidant capacities have shown promise, leading to hypotheses of the importance of foods with high antioxidant composition in the prevention of apoptosis and neuronal cell death in neurodegenerative diseases like AD [11,12]. In the current study, we have been able to demonstrate that the ethyl acetate extract of GBR has high antioxidant capacity and also possesses anti-apoptotic and pro-survival properties in neuronal cells.

## Conclusions

Germination of BR is known to improve its bioactive compounds and several studies have reported enhanced functional effects of GBR over BR. Also, GBR has been shown to possess high antioxidant capacity, as we have demonstrated in the current study. Interestingly, oxidative damage on neurons is mediated mostly through apoptosis, and dietary components with high antioxidant capacities have been shown to prevent or at least delay the processes leading up to neurodegeneration. In the current study, we showed that the ethyl acetate extract of GBR increased cell viability in the presence of H_2_O_2_. The increase in cell viability was likely mediated through anti-apoptotic effects of the extract as demonstrated by the morphological changes under microscope and changes in transcriptional regulation of genes that tended more towards survival and less towards apoptosis. Taken together, it could be argued that GBR may reduce oxidative stress-induced neurotoxicity due to its high antioxidant potentials and ability to promote neuronal cell survival in the presence of H_2_O_2_-induced oxidative stress, since oxidative stress is an important contributor to neurodegenerative diseases. Our findings are therefore worth studying further, in order to provide more insight into the protective effects of GBR and its bioactives against neurodegenerative disease processes like AD, and especially what regulatory pathways they modulate.

## Abbreviations

AD: Alzheimer’s disease; ABTS: 2,2′-azino-bis[3-ethylbenzothiazoline-6-sulphonic acid]; DPPH: Di[phenyl]-[2,4,6-trinitrophenyl]iminoazanium; GAE: Gallic acid equivalents; H2O2: Hydrogen peroxide; MTT: 3-(4,5-Dimethylthiazol-2-yl)-2,5-diphenyltetrazolium bromide; DMSO: Dimethyl sulfoxide; GBR: Germinated brown rice; BR: Brown rice; NF-Kβ: Nuclear factor-kappa β; PBS: Phosphate buffered saline; ROS: Reactive oxygen species; TEAC: Trolox Equivalents; MAPK: Mitogen-activated protein kinase; JNK: Jun N-terminal kinase; ERK: Extracellular signal related kinases; COX: Cyclooxygenase; GAPDH: Glyceraldehyde 3-phosphate dehydrogenase.

## Competing interests

The authors declare that they have no competing interests.

## Authors’ contributions

NHA designed the study and carried out the experimental parts. MUI critically read and revised the manuscript. NI and MI supervised the work and experimental designs, and approved the final manuscript for submission. All authors read and approved the final manuscript.

## Pre-publication history

The pre-publication history for this paper can be accessed here:

http://www.biomedcentral.com/1472-6882/13/177/prepub
